# Emotional Eating and Abdominal Obesity: A Narrative Review of the Potential Mechanisms Underlying Their Relationship and Emerging Interventions for Their Management

**DOI:** 10.3390/nu18111767

**Published:** 2026-05-30

**Authors:** Leslie Yunuén Guillén-Medina, Norma Patricia Rodriguez-Rocha, Martha Betzaida Altamirano-Martínez, Gabriela Maldonado-Ulloa, Greissy Vianey Mora-López, Gabriela Macedo-Ojeda

**Affiliations:** 1 Departamento de Alimentación y Nutrición, Centro Universitario de Ciencias de la Salud, Universidad de Guadalajara, Sierra Mojada 950, Independencia, Guadalajara 44340, Jalisco, Mexico; leslie.guillen9862@alumnos.udg.mx (L.Y.G.-M.); norma.rodriguez@academicos.udg.mx (N.P.R.-R.); martha.altamirano@academicos.udg.mx (M.B.A.-M.); gabriela.maldonado9440@alumnos.udg.mx (G.M.-U.); greissy.mora9439@alumnos.udg.mx (G.V.M.-L.); 2 Instituto de Investigación en Ciencias Biomédicas, Centro Universitario de Ciencias de la Salud, Universidad de Guadalajara, Sierra Mojada 950, Independencia, Guadalajara 44340, Jalisco, Mexico

**Keywords:** emotional eating, abdominal obesity, physiological mechanisms, psychological mechanisms, hedonic hunger

## Abstract

Abdominal obesity (AO), assessed through waist circumference (WC), has become a validated complementary anthropometric marker for cardiometabolic risk assessment. Growing evidence suggests that emotional eating (EE), characterized by food intake in response to emotions rather than physiological hunger cues, may be linked to AO by promoting dysregulated eating patterns rich in palatable and energy-dense foods. This behavior may contribute, directly or indirectly, to excess visceral fat accumulation. An analysis of the current evidence was conducted to examine the psychological, physiological, neuroendocrine, and gut–brain axis mechanisms underlying the association between EE and AO, as well as to explore emerging interventions for its management. A narrative review of studies published between 2015 and 2025 was carried out using PubMed and Google Scholar. Search terms related to EE, AO, physiological mechanisms, hedonic hunger, diet quality, gut microbiota, and mindful eating were employed. The findings indicate that EE is associated with emotional dysregulation, chronic activation of the hypothalamic–pituitary–adrenal (HPA) axis, increased cortisol levels, low-grade inflammation, alterations in neurotransmitters such as dopamine, serotonin, and neuropeptide Y (NPY), as well as intestinal dysbiosis. These mechanisms favor impulsive consumption of palatable foods, visceral fat gain, and metabolic deterioration. Interventions such as mindful eating show positive effects in reducing EE; however, their anthropometric impact still requires further validation. In conclusion, EE represents an important behavioral factor in the development and maintenance of AO. Its management requires a multidimensional approach integrating emotional regulation, dietary modification, and psychobehavioral strategies.

## 1. Introduction

Abdominal obesity (AO) is defined as excessive fat accumulation in the abdominal region, assessed by waist circumference (WC), which varies according to sex and ethnicity. According to the World Health Organization (WHO), AO is defined in Europe, South America, and North America as a WC ≥ 94 cm in men and ≥80 cm in women, whereas in the Middle East, South and East Asia, similar but slightly lower cutoff values are applied (≥90 cm for men and ≥80 cm for women) due to differences in ethno-racial risk profiles [1]. In contrast, in the United States, the National Heart, Lung, and Blood Institute (NHLBI) establishes that a WC ≥ 102 cm in men and ≥88 cm in women indicates AO [2].

The prevalence of AO has increased significantly [3,4]. Substantial evidence supports the association between abdominal fat accumulation and an increased risk for metabolic conditions such as dyslipidemia, type 2 diabetes, hypertension, and cardiovascular diseases, positioning AO as a more precise indicator of cardiometabolic risk related to excess adiposity [5,6,7]. Importantly, current guidelines recommend using body mass index (BMI) primarily as a population-level health risk assessment tool, suitable for epidemiological studies or screening purposes, rather than as an individual measure of health [8]. This highlights the need for complementary, validated anthropometric assessments, such as the WC, to more accurately evaluate cardiometabolic risk.

Substantial evidence supports the association between abdominal fat accumulation and an increased risk for metabolic conditions such as dyslipidemia, type 2 diabetes, hypertension, and cardiovascular diseases, positioning AO as a more precise indicator of cardiometabolic risk related to excess adiposity [6,7,8]. Current evidence suggests that WC should be considered a clinical vital sign because it provides additional prognostic information beyond BMI and more accurately reflects visceral adiposity and cardiometabolic risk [9].

Research indicates a potential association between EE and AO [10,11,12,13]. EE is generally defined as the tendency to eat in response to emotional states, including both negative and positive emotions [14,15,16,17]. This behavior typically emerges in contexts of emotional regulation dysfunction, chronic stress, anxiety, or depression, which may activate the hypothalamic–pituitary–adrenal (HPA) axis and increase cortisol levels, thereby promoting the consumption of comfort foods [18,19].

Previous evidence suggests that EE may function as a maladaptive emotion-regulation strategy associated with stress vulnerability, depressive symptoms, and increased susceptibility to weight gain and obesity [20].

In parallel, shared pathophysiological mechanisms between EE and AO have been described, including chronic low-grade inflammation, insulin resistance, and alterations in appetite-regulating hormonal signaling [7,21]. Recent studies have also suggested a potential role of neurotransmitters and reward-related neural circuits in modulating hedonic eating behavior, particularly under stress conditions [22,23,24]. Hedonic hunger is closely related to EE, as both describe intake patterns driven by non-homeostatic factors, independent of physiological energy needs where the consumption of palatable and energy-dense foods is motivated by pleasure, emotional relief, or affective regulation rather than by metabolic hunger or satiety signals [22]. Indeed, a significant correlation has been observed between hedonic hunger, emotional dysregulation, and increases in BMI [22,25].

Additionally, the gut–brain axis, through the intestinal microbiota, its metabolites (such as short-chain fatty acids, indoles, and secondary bile acids), and its influence on the secretion of orexigenic and anorexigenic hormones, represents an emerging component in this bidirectional interaction between EE and AO [26,27]. Alterations in the composition and function of the gut microbiota may promote both visceral fat accumulation and changes in EE behavior through neuroinflammatory mechanisms and modulation of the reward system [28,29].

Furthermore, evidence suggests that EE may negatively influence diet quality by favoring the consumption of energy-dense, nutrient-poor foods, such as calorie-dense snacks and fast food, particularly in response to negative emotions [11,30]. This pattern is especially relevant in individuals with AO, who also tend to have lower intake of fruits, vegetables, and dietary fiber [11], potentially contributing to the persistence of excess adiposity and increased metabolic vulnerability. In this context, EE emerges as a key behavioral determinant influencing both food selection and the risk of AO.

Through a review of recent studies, the present work aims to summarize the current evidence on the potential mechanisms associating EE with AO. In addition, specific intervention strategies investigated for addressing EE in this population will be discussed.

## 2. Materials and Methods

### 2.1. Search Strategy

A structured narrative literature search was conducted in PubMed, Scopus, Web of Science, Embase, PsycINFO, and Google Scholar to identify studies published between January 2015 and March 2025 related to emotional eating (EE), abdominal obesity (AO), and their underlying mechanisms. The search strategy combined controlled vocabulary terms and free-text keywords using Boolean operators (“AND”, “OR”) and database-specific adaptations.

The search combined terms related to “emotional eating”, “emotional overeating”, “abdominal obesity”, “waist circumference”, “visceral adiposity”, “central obesity”, “stress”, “anxiety”, “depression”, “cortisol”, “neurotransmitters”, “hedonic hunger”, “gut-brain axis”, “microbiota”, “diet quality”, “mindfulness”, and “mindful eating”. Database filters were applied when available, including publication dates between 2015 and 2025, studies conducted in humans, articles published in English or Spanish, and document types restricted to original research articles, systematic reviews, meta-analyses, and narrative reviews. The inclusion criteria were: (i) original research articles, systematic reviews, meta-analyses, and narrative reviews providing empirical, conceptual, or mechanistic evidence related to EE and AO; (ii) studies conducted in adults or adolescents evaluating abdominal obesity, waist circumference, visceral adiposity, emotional eating, diet quality, neurobiological mechanisms, gut microbiota, or mindfulness-based interventions; (iii) articles published in English or Spanish; and (iv) studies published between 2015 and 2025.

Seminal studies published before 2015 were additionally included when considered essential for conceptual or mechanistic understanding. The exclusion criteria were: (i) studies unrelated to the objectives of the review; (ii) studies focused exclusively on general obesity without assessment of abdominal adiposity or waist-related indicators, unless they provided highly relevant mechanistic evidence; (iii) conference abstracts, editorials, letters to the editor, dissertations, commentaries, and gray literature; (iv) duplicate publications; and (v) articles with insufficient methodological detail, inadequate outcome reporting, or limited relevance to the thematic synthesis.

To reduce selection bias, predefined eligibility criteria were established before the screening process and consistently applied throughout study selection.

### 2.2. Research Question

The present narrative review aimed to provide a comprehensive overview of the current scientific evidence regarding the relationship between EE and AO, integrating psychological, physiological, neuroendocrine, and gut–brain axis mechanisms, as well as emerging behavioral interventions for their management.

Accordingly, the guiding research question was: what psychological, physiological, and neurobiological mechanisms explain the association between EE and OA, and what evidence exists regarding behavioral interventions for their management?

### 2.3. Study Selection and Data Extraction

The study selection process consisted of an initial screening of titles and abstracts, followed by full-text assessment of potentially eligible studies according to the predefined inclusion and exclusion criteria conducted independently by a single author.

For each included study, the following information was extracted: (i) author and year of publication; (ii) study population and sample size; (iii) study design; (iv) variables assessed, including EE, waist circumference, visceral adiposity, dietary patterns, neurobiological markers, or psychological variables; and (v) principal findings related to the association between EE and AO.

The extracted data were subsequently organized into thematic categories to facilitate narrative synthesis and interpretation of the evidence.

### 2.4. Data Synthesis

Given the broad and multidimensional scope of the topic, evidence from original studies, systematic reviews, meta-analyses, and narrative reviews was considered to achieve a comprehensive understanding of the complex interactions underlying EE and AO.

Due to the heterogeneity of study designs, populations, methodologies, and assessed outcomes, a quantitative meta-analysis was not performed. Instead, a qualitative narrative synthesis was conducted to provide a comprehensive overview of the available evidence and identify converging mechanistic pathways, behavioral patterns, and emerging therapeutic approaches.

The evidence was organized into thematic domains to facilitate interpretation and provide a comprehensive overview of the current understanding of: (i) the conceptualization and assessment of EE; (ii) the association between EE and AO; (iii) psychological mechanisms; (iv) physiological and neuroendocrine mechanisms; (v) gut–brain axis interactions; (vi) diet quality and eating behavior; and (vii) behavioral interventions such as mindfulness and mindful eating.

The literature search and selection process identified 120 scientific publications considered relevant to the objectives of this narrative review.

## 3. Results

### 3.1. Conceptualization of Emotional Eating

The concept of EE can be traced back to the psychosomatic theory of obesity proposed by Kaplan and Kaplan, which suggested that overeating may occur as a learned response to anxiety or emotional distress [31]. This perspective was later expanded by Bruch, who proposed that some individuals may have difficulty distinguishing physiological hunger from internal emotional states [32]. Alongside these early explanations, restraint theory suggested that cognitive attempts to restrict food intake may increase vulnerability to overeating when dietary control is disrupted, a process commonly described as disinhibition [33]. Together, these perspectives provide foundational explanations for why eating may become detached from physiological hunger and instead be guided by affective states.

In contemporary literature, EE is generally defined as eating in response to negative emotions such as stress, sadness, and anxiety; however, some authors suggest that it may also occur in response to positive emotions such as joy [14,15,16,17,34]. As a core feature of EE, eating in response to negative emotions reflects a disruption in the regulation of food intake by homeostatic signals, such as hunger and satiety. This pattern may be partly explained by difficulties in differentiating bodily hunger and fullness cues from the physiological manifestations of emotional arousal [30].

According to Macht’s five-way model of emotion and eating, emotions can influence food intake through multiple pathways, including emotional control of eating, suppression of intake under acute stress, impairment of cognitive eating control, and emotion-congruent modulation of food choice [34].

**Figure 1:**
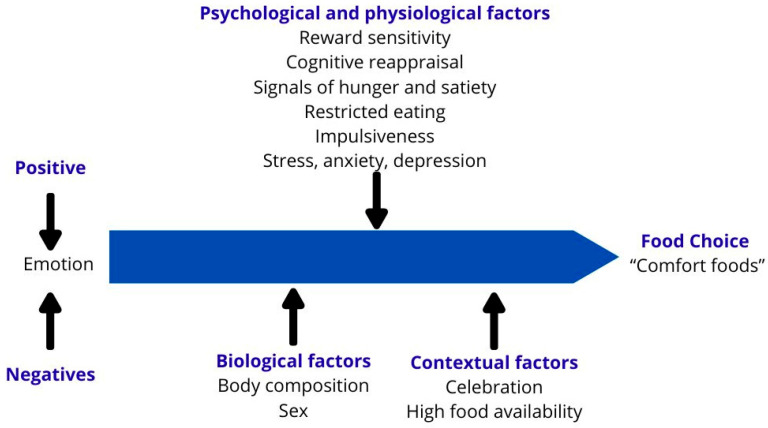
Multidimensional Determinants of Emotional Hunger and Food Choice. Current evidence on the emotional, psychological, physiological, biological, and contextual determinants of emotional hunger. The figure illustrates the interaction of emotional, psychological, physiological, and contextual factors in food choice. Positive and negative emotions influence eating decisions, while psychological factors such as reward sensitivity, stress, and depression, together with physiological signals of hunger and satiety, play a crucial role. Biological elements, including body composition and sex, as well as contextual factors such as celebrations and food availability, are also considered, highlighting the complexity and multidimensional nature of eating decisions across different contexts and emotional states. Figure created using graphic resources from Canva Pro^®^ (Canva Pty Ltd., Sydney, Australia; https://www.canva.com/; accessed on 28 April 2026).

Consistent with this multidimensional view, some researchers have proposed that emotional hunger is influenced by psychological, biological, contextual, and emotional determinants (Figure 1) [34,35].

This behavior is frequently associated with the selection of unhealthy and ultra-processed foods (UPFs) and snacks with high energy density, high sugar content, and high fat content, commonly perceived as palatable and comforting foods [14,36,37,38,39,40,41,42]. Additionally, individuals with EE, particularly those with AO, show a lower intake of fruits, vegetables, and dietary fiber, reflecting a reduced adherence to healthy dietary patterns [11].

The term “comfort food” refers to foods that, when consumed, provide comfort or a sense of satisfaction by offering some degree of psychological relief [43]. Research on comfort foods and EE reveals complex relationships between emotions, eating behavior, and food preferences. Emotional eaters tend to consume larger amounts of food when experiencing negative emotions, and palatability seems to mediate the relationship between food intake and mood improvement [30]. In women, comfort food consumption is often triggered by negative affect, whereas in men it is more frequently motivated by positive emotions [44].

Evidence suggests that comfort eating, especially the consumption of sweet and high-fat foods, may provide a transient improvement in mood but can also have negative consequences for body composition [43]. These and other effects of EE have been associated with the development of several health conditions, including obesity, eating disorders, diabetes, and cardiovascular diseases [45].

#### Evaluation of Emotional Eating

The validity of the concept of EE is controversial, with mixed evidence from psychometric, experimental, and naturalistic studies; however, it is consistently observed in individuals with binge eating disorder [16].

Recent research has questioned both the conceptual validity of EE as a construct and the accuracy of self-report measures. While some studies support the presence of EE in individuals with eating disorders [16], the evidence in healthy populations remains inconsistent. Laboratory studies have failed to find an association between self-reported EE and actual food intake during negative mood inductions [46]. Some researchers argue that EE scales lack predictive and discriminant validity, suggesting alternative interpretations such as concerned eating or cue-reactive eating [47]. Nevertheless, a recent study integrating laboratory-based, psychometric, and daily-life measures provided support for a unified EE construct that correlates with eating disorder symptoms and BMI, but not with restrained eating [48]. These contradictory findings highlight the complexity of EE and underscore the need for further research employing diverse methodological approaches to improve the understanding of this phenomenon.

### 3.2. Emotional Eating and Abdominal Obesity

Several studies have demonstrated that EE is positively associated with WC, a marker of cardiometabolic risk (Table 1). In Finnish adults, EE was found to mediate the relationship between depressive symptoms and increases in WC over a seven-year follow-up period [13]. Consistently, studies conducted in Turkish and Slovenian adults reported positive associations between EE, WC, and AO [10,12]. Although the overall association was negative in Greek adults, it became positive among individuals older than 35.9 years [49].

Similarly, studies involving Polish university students and Argentine adults observed associations between EE, BMI, and the waist-to-height ratio (WHtR) [51,52]. Taken together, the evidence highlights EE as a factor associated with greater abdominal adiposity, with differences according to age and sex.

#### 3.2.1. Emotional Eating and Abdominal Obesity: Mechanisms Underlying Their Association

AO and EE are interconnected through complex psychological and physiological mechanisms. Complex psychological and physiological mechanisms have been proposed to underlie the association between AO and EE such as impaired emotion regulation and elevated levels of stress and anxiety, which may contribute to behaviors such as EE. However, this relationship may also be bidirectional, as emotional distress can trigger EE even in individuals without obesity, potentially leading to increased energy intake and subsequent weight gain. These behaviors represent responses to negative emotions and often result in the consumption of unhealthy foods, thereby perpetuating a cycle of body fat gain and emotional distress [53].

In addition, dysregulation of physiological mechanisms—including immune-inflammatory processes, hormonal alterations, and dysfunction of the HPA axis—has been proposed as a potential link between AO and EE [54].

##### Psychological Factors and Physiological Mechanisms Related to Emotional Eating

EE is associated with several psychological factors and adverse health outcomes. Current evidence indicates that depression, anxiety, and stress are often linked to increased levels of EE behaviors [55,56].

Research further supports a robust association between stress and EE. Perceived stress has been associated with increased EE, particularly among women [57]. When individuals experience stress, the HPA axis is activated, accompanied by increased cortisol release, one of the primary stress hormones [18]. Elevated glucocorticoids, particularly cortisol, have been associated with a greater intake of “comfort foods” high in sugar and fat, which may temporarily reduce stress-related negative emotions [19,58]. Stress-related eating behaviors are mediated by the brain’s opioid and dopaminergic systems, as well as the serotonergic system in vulnerable individuals [59]. Chronic stress and sustained elevations in glucocorticoids have been associated with visceral fat accumulation, commonly related to AO and associated metabolic abnormalities collectively referred to as metabolic syndrome [19]. Depression has similarly been associated with chronic hyperactivation of the HPA axis with elevated cortisol levels, which may increase the risk of AO and enhance neurocognitive signals associated with hedonic hunger, thereby fostering impulsive or EE behaviors [60].

Similar to stress, anxiety has been associated with HPA axis activation [61], which, in combination with impulsivity, may lead to the consumption of high-fat and high-sugar foods as a mechanism for temporary relief [62,63]. This process increases the risk of abdominal fat accumulation. It has been proposed that individuals with anxiety exhibit amplified activation of reward-related neural circuits in response to food cues, predisposing them to seek highly palatable foods to alleviate emotional distress and reinforcing the cycle of EE [53]. Evidence further suggests that individuals with generalized anxiety disorder (GAD) may engage in EE as a strategy to avoid negative emotions, with emotional dysregulation playing a central role [64].

It has been reported that adults with overweight or obesity who engage in EE in response to depression, anxiety/anger, and boredom exhibit poorer psychological well-being, greater eating disorder symptomatology, and more pronounced difficulties in emotional regulation. In contrast, eating in response to positive emotions was not significantly associated with these outcomes [55].

In summary, EE represents a potential pathway through which stress, anxiety, and depression negatively influence eating behavior and metabolic health. Factors such as HPA axis hyperactivity, impulsivity, and psychological inflexibility promote the consumption of highly palatable foods as a strategy for emotion regulation, thereby increasing the risk of AO.

AO is characterized by chronic low-grade inflammation [65], which may contribute to emotional distress and behavioral symptoms [53,66]. The accumulation of free fatty acids (FFAs) and reactive oxygen species (ROS) in adipose tissue (AT) stimulates the release of pro-inflammatory adipokines, including leptin, resistin, tumor necrosis factor alpha (TNF-α), interleukin-6 (IL-6), and chemerin, which contribute to insulin resistance [7]. This process promotes immune system activation, resulting in chronic low-grade inflammation [7]. Associations have been reported between inflammatory markers such as C-reactive protein (CRP) and IL-6 with obesity-related eating behaviors, including loss of control eating [21]; however, available evidence remains limited.

Some studies have linked AO to neuroendocrine alterations, particularly dysfunction of the HPA axis. Chronic activation of the HPA axis leads to elevated and sustained cortisol levels. Although cortisol is essential for systemic adaptation to stress, excessive exposure promotes visceral adipose tissue accumulation, insulin resistance, and chronic inflammation [67]. At the molecular level, excess cortisol enhances gluconeogenesis, induces peripheral insulin resistance, and disrupts the regulation of immune and inflammatory systems [67]. Some studies have linked AO to hormonal alterations, particularly dysfunction of the HPA axis. However, one study did not find a significant association between cortisol levels and risky eating behaviors, such as EE or food addiction [68].

Moreover, evidence supports a significant relationship between hedonic hunger, EE, and adiposity, although longitudinal studies are still required to establish clearer causal links. A meta-analysis reported a positive but small effect size association (r = 0.13; 95% CI: 0.08–0.18) between hedonic hunger and BMI [25]. Another study found that EE was associated with an increased risk of weight gain and obesity, as it promotes a sustained positive energy balance [69]. Additionally, greater difficulties in emotion regulation have been correlated with higher levels of both hedonic hunger (r = 0.325; *p* < 0.001) and BMI (r = 0.176; *p* < 0.001) [22]. Collectively, these findings suggest that emotional dysregulation may mediate the relationship between pleasure-driven eating and body adiposity.

**Figure 2:**
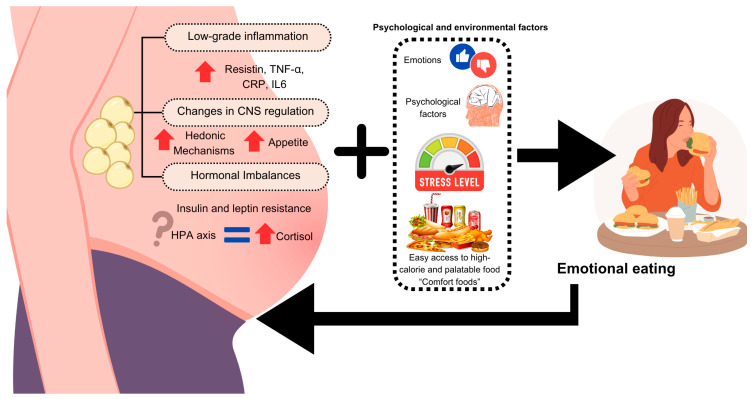
Bidirectional links between abdominal obesity and emotional eating. This figure summarizes proposed mechanisms underlying the relationship between physiological alterations and psychological and environmental factors associated with AO and EE. The left side illustrates potential effects of AO, including low-grade inflammation characterized by increased levels of inflammatory markers such as resistin, TNF-α, C-reactive protein (CRP), and IL-6; alterations in central nervous system (CNS) regulation that enhance hedonic mechanisms and appetite; as well as hormonal imbalances related to insulin and leptin resistance, and a potential increase in cortisol levels due to hyperactivation of the hypothalamic–pituitary–adrenal (HPA) axis. Red upward arrows indicate increased levels, activation, or upregulation of the indicated markers or processes. The question mark indicates uncertainty or limited direct evidence regarding the proposed pathway between HPA-axis activity and cortisol alterations in the context of AO and EE. The “=” symbol is used to represent a proposed association between HPA-axis dysregulation and increased cortisol levels, rather than a confirmed causal relationship. However, several of these proposed pathways, particularly those linking inflammatory processes with EE, are supported by limited direct evidence in humans and are derived largely from preclinical or indirect evidence. On the right, emotional and psychological factors, stress, and availability of hypercaloric and highly palatable foods are highlighted as potential triggers of EE. Black arrows represent the current hypothesis of a bidirectional relationship between AO and EE. CRP: C-reactive protein; HPA: hypothalamic–pituitary–adrenal; IL-6: interleukin-6; TNF-α: tumor necrosis factor alpha. Figure created using graphic resources from Canva Pro^®^ (Canva Pty Ltd., Sydney, Australia; https://www.canva.com/; accessed on 28 April 2026).

Individuals exhibiting EE patterns may therefore be at increased risk of abdominal fat accumulation [10,52]. Furthermore, individuals with EE tend to exhibit elevated cortisol concentrations [60], which may perpetuate metabolic dysfunction. Accordingly, AO and EE are considered to share a bidirectional relationship (Figure 2).

##### Role of Neurotransmitters and Hormones

Neurotransmitters and hormones have been reported to play a crucial role in the regulation of appetite, AO and EE through complex central and peripheral signaling systems (Table 2). Within the hypothalamus, the arcuate nucleus contains two main neuronal populations that regulate appetite: catabolic neurons expressing pro-opiomelanocortin (POMC) and anabolic neurons synthesizing neuropeptide Y (NPY) and agouti-related peptide (AgRP) [23]. Evidence further suggests that glutamate, gamma-aminobutyric acid (GABA), dopamine, serotonin (5-HT), orexin, leptin, ghrelin, and NPY also play key roles in the regulation of adiposity and EE [23,70].

##### Homeostatic Dysregulation and Energy Balance

POMC regulates satiety and energy expenditure through melanocortin signaling, activating catabolic pathways that reduce food intake [71]. Impaired mitochondrial and inflammatory signaling in POMC neurons may disrupt their ability to regulate appetite and energy expenditure, potentially contributing to increased food intake and reduced energy expenditure [72].

Lower levels of POMC in cerebrospinal fluid (CSF) have been reported in individuals with overweight and obesity compared with normal-weight individuals. Moreover, a negative correlation has been observed between CSF and POMC levels with body fat percentage, suggesting that higher adiposity may be associated with lower POMC levels [73]. These metabolic alterations may contribute to the association between AO and EE, as impaired satiety signaling and reduced energy expenditure can facilitate overeating behaviors. Under emotionally stressful conditions, these factors may increase susceptibility to EE and subsequently promote visceral fat accumulation [72].

In conjunction with psychological factors such as stress, these neurobiological alterations may contribute to EE. Preclinical studies in animal models have shown that chronic stress can induce hyperactivity of hypothalamic POMC neurons, impair neuronal plasticity, and promote emotional-like symptoms. These alterations may provide a plausible mechanistic link between stress, appetite dysregulation, EE, and a potentially increased risk of AO. However, their direct translation to humans requires further investigation [74].

NPY is a potent orexigenic peptide involved in appetite stimulation, food-seeking behavior, energy expenditure, and metabolic regulation [23,75]. In obesity-related contexts, dysregulation of NPY signaling may favor increased appetite, reduced energy expenditure, and fat accumulation. In addition, NPY interacts with dopaminergic and serotonergic reward pathways, which may contribute to hedonic eating and preference for energy-dense foods [78]. Therefore, stress-induced increases in NPY activity may constitute a mechanism linking EE and AO, since an enhanced preference for highly palatable foods can contribute to excessive caloric intake and abdominal fat accumulation [58,60].

In preclinical animal models, AgRP neuron activation has been associated with fat accumulation and increased motivation for energy-dense foods, suggesting a possible mechanism involved in stress- or emotion-related eating. However, direct evidence linking AgRP neuronal activity to EE in humans remains limited [79]. Beyond regulation intake based on energy needs [80], these neurons seem to increase motivation toward highly rewarding foods by acting on brain regions such as the paraventricular thalamus and lateral hypothalamus. Inhibition of aversive signals in the parabrachial nucleus may reinforce this behavior, prioritizing the consumption of pleasurable, energy-dense foods [81,82],. Additionally, AgRP neuron activation has been reported to modulate food-motivated learning and emotional responses related to food availability, integrating hunger regulation with emotional processing [83].

Leptin regulates satiety by inhibiting orexigenic neurons and activating anorexigenic neurons in the hypothalamus, which could lead to decreased motivation towards rewarding foods [96]. In obesity, leptin resistance weakens these regulatory mechanisms, promoting overeating and visceral fat accumulation [97]. This dysfunction may further increase susceptibility to EE, particularly under stress conditions, where glucocorticoids exacerbate leptin resistance. This impaired satiety signaling may contribute to the association between AO and EE by increasing susceptibility to emotionally driven overeating behaviors [58].

Ghrelin is an orexigenic hormone that stimulates food intake through activation of AgRP/NPY neurons in the hypothalamus [98]. It has also been linked to alterations in dopamine signaling that may influence motivation toward reward food [99]. Ghrelin has been associated with fat accumulation through mechanisms involving increased food intake, reduced energy expenditure, and activation of AgRP/NPY neurons and intracellular pathways [100]. Under stress conditions, ghrelin has also been implicated in appetite alterations induced by HPA axis–mediated appetite suppression. Through these mechanisms, ghrelin may strengthen the association between emotional stress, emotional eating behaviors, and the development of abdominal obesity [58].

Insulin has been implicated in the regulation of food intake and body weight through mechanisms involving inhibition of AgRP/NPY neurons and stimulation of POMC neurons in the hypothalamus [81,101], as well as reducing motivation for palatable foods through the reward system [102]. In obesity, central insulin resistance has been associated with alterations in these regulatory mechanisms, which may contribute to visceral fat accumulation and increased susceptibility to EE, particularly when resistance develops in regions such as the central amygdala. These alterations may elucidate the mechanisms by which obesity-related metabolic dysfunction contributes to EE behaviors that perpetuate visceral fat accumulation and AO [103].

##### Reward-Related and Emotional Pathways

Recent studies have consistently linked elevated circulating glutamate levels with obesity, particularly abdominal fat accumulation [84,85]. Plasma glutamate concentrations show an inverse association with adiponectin levels in both patients with type 2 diabetes and obese mouse models [86]. In female mice, a glutamatergic circuit connecting the paraventricular thalamic nucleus to the insular cortex has been identified as a mediator of stress-induced compulsive eating, without affecting other feeding behaviors. This interaction between homeostatic and emotional signals may help explain how dysregulated eating behaviors associated with EE could contribute to the development or maintenance of AO [87].

GABA released by NPY/AgRP neurons in the arcuate nucleus inhibits anorexigenic activity in the parabrachial nucleus, suppressing satiety and aversion signals and allowing behavioral and metabolic adaptation to energy deficits [88]. GABA has been shown to inhibit body weight gain, suppress adipogenesis, and improve lipid profiles in mice with diet-induced obesity. [89]. However, activation of GABAergic neurons in the ventral tegmental area (VTA) is linked to anxiety-like behaviors and excessive intake of hyperpalatable foods [90]. Alterations in these pathways may therefore represent a potential mechanism by which emotional and reward-related processes influence eating behaviors associated with AO and EE.

Serotonin (5-HT) acts on 5-HT2C receptors in POMC neurons to regulate food intake and energy expenditure [23]. It also modulates motivation toward palatable foods through its actions in the VTA and nucleus accumbens (NAc), thereby interfering with emotionally driven food seeking [91]. In obesity, reduced serotonergic signaling within these circuits diminishes appetite control and increases the drive toward rewarding foods [24], promoting EE and abdominal fat accumulation. This dysfunction may be exacerbated by high-fat and high-sugar diets, which lead to a reduction in hypothalamic serotonin release; consequently, impaired serotonergic signaling may facilitate the interaction between EE and AO by enhancing emotionally driven food seeking and prolonged consumption of highly rewarding foods [91].

Oxytocin is both a neurotransmitter and hormone involved in appetite regulation [23]. It is linked with satiety by activating POMC neurons and inhibiting AgRP/NPY neurons in the hypothalamus [92]. Oxytocin has been associated with motivation toward palatable foods by modulating reward-related brain regions [93]. Regarding adiposity, oxytocin has been associated with browning of white adipose tissue, stimulates thermogenesis, and enhances lipolysis [94], which may be related to reductions in visceral fat. Additionally, oxytocin has been linked with EE by reducing activation of food-motivation circuits and enhancing activity in cognitive control regions [95]. These findings indicate that oxytocin-related pathways could attenuate mechanisms underlying both EE and AO by modulating appetite regulation and emotional responses to food.

Taken together, these pathways indicate that the interplay among disrupted satiety signaling, altered reward processing, and stress-related eating behaviors may contribute to a bidirectional relationship between EE and AO. Emotional overeating may contribute to visceral adiposity, while neurobiological changes associated with obesity may further reinforce EE behaviors.

##### Gut–Brain Axis

**Figure 3:**
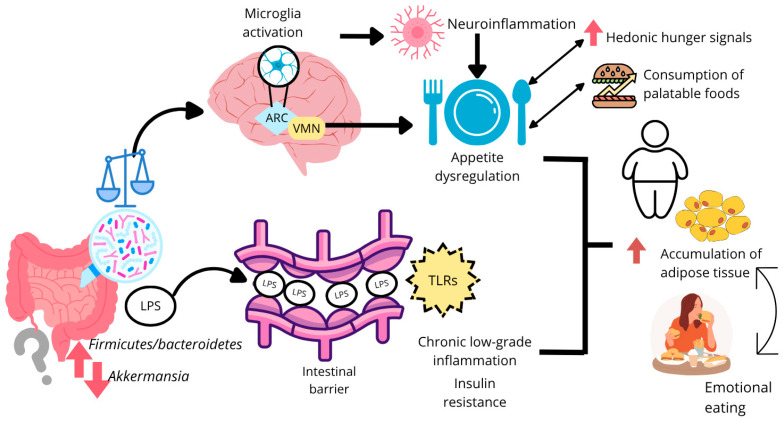
Relationship between the gut–brain axis, AO, and EE. The figure illustrates how an imbalance in the gut microbiota composition, including changes in the *Firmicutes*/*Bacteroidetes* ratio and reduced *Akkermansia* abundance, may contribute to increased intestinal permeability and lipopolysaccharide (LPS) translocation, which have been associated with activation of Toll-like receptors (TLRs), chronic low-grade inflammation, and insulin resistance. These thereby facilitate adipose tissue accumulation. Simultaneously, LPS can induce microglial activation and neuroinflammation in key brain regions such as the arcuate nucleus and the ventromedial nucleus (VMN), which have been linked to appetite dysregulation characterized by enhanced hedonic hunger signaling and increased consumption of highly palatable foods. These alterations may be associated with AO, which in turn reinforces EE, suggesting a potentially self-reinforcing interplay among metabolic alterations, inflammation, eating behavior, and the gut microbiota. Black arrows indicate proposed pathways or directions of association between mechanisms. Bidirectional black arrows indicate potentially reciprocal interactions. Red upward arrows indicate increased levels, abundance, activation, or accumulation, whereas red downward arrows indicate reduced abundance or decreased levels. The question mark indicates uncertainty or limited direct evidence regarding the specific role of gut microbiota alterations in the AO–EE relationship. Figure created using graphic resources from Canva Pro^®^ (Canva Pty Ltd., Sydney, Australia; https://www.canva.com/; accessed on 28 April 2026).

The gut–brain axis seems to play a crucial role in the development of obesity (Figure 3) through bidirectional communication involving neural, endocrine, and inflammatory pathways [104,105]. The composition of the gut microbiota, influenced by factors such as diet and environmental exposures, may affect metabolism, appetite regulation, and insulin sensitivity [105]. The gut microbiota modulates the secretion of orexigenic hormones (e.g., ghrelin) and anorexigenic hormones (e.g., leptin, PYY, glucagon-like peptide-1 (GLP-1), and cholecystokinin (CCK) through microbial metabolites such as SCFAs, indoles, and secondary bile acids, which act on host receptors including G protein–coupled receptors (GPRs) and the nuclear farnesoid X receptor (FXR) [26,27,104]. In addition, SCFAs—particularly acetate, butyrate, and propionate—activate free fatty acid receptors FFAR2 and FFAR3 in the intestine, stimulating the release of leptin, GLP-1, and PYY [27]. Certain SCFAs can cross the blood–brain barrier and directly act on hypothalamic neurons, POMC expression, and inhibit neuropeptide Y/agouti-related peptide (NPY/AgRP) signaling [27,106,107]. Collectively, these mechanisms contribute to the regulation of satiety, lipid metabolism, and visceral fat storage [26].

Gut bacteria also produce or regulate neurotransmitters such as 5-HT, dopamine, glutamate, GABA, and histamine, which may influence both the homeostatic and emotional regulation of appetite and body fat accumulation [28,108,109]. For example, microbiota-induced serotonin acts on POMC neurons to promote satiety [28].

Some studies have reported alterations in gut microbiota composition in individuals with obesity, including changes in the *Firmicutes*-to-*Bacteroidetes* ratio. However, recent evidence has questioned the reproducibility and consistency of this ratio as a robust obesity-related microbial signature across populations and methodologies [110,111]. Nevertheless, obesity-associated dysbiosis may contribute to enhanced dietary energy harvest and increased adipose tissue accumulation [26,112,113,114], including abdominal fat. Alterations in gut microbial ecosystems promote the production of bacterial endotoxins, such as LPS, which translocate across a compromised intestinal barrier, activate Toll-like receptors (TLRs), and contribute to chronic low-grade inflammation and the development of insulin resistance, thereby favoring abdominal fat accumulation [26,109,115,116]. An altered microbiota composition may also contribute to neuroinflammation through microglial activation in brain regions such as the NAc, disrupting reward signaling and facilitating hyperphagic behaviors [28,109,117].

The gut microbiome seems to play a critical role in the regulation of eating behavior and emotions through the gut–brain axis [117]. It modulates neural circuits involved in appetite regulation, particularly via the vagus nerve, affecting the activity of the arcuate nucleus and the VTA. This mechanism is central to hedonic responses to food, influencing both “wanting” and “liking” of palatable foods [27,28]. Evidence suggests that gut microbiota composition influences brain reward circuitry, thereby affecting behavior and emotional states [117,118,119,120]. Dysbiosis may induce inflammatory responses and negative emotional states [121], which together may contribute to both AO and EE.

A study in individuals with obesity and binge eating behavior disorder identified specific microbial signatures, including reduced abundance of *Akkermansia* and *Intestinimonas* and increased levels of *Anaerostipes* and *Bifidobacterium*, which may contribute to EE through neuroactive metabolites modulation and the gut–brain axis regulation [122]. Another study in murine models and humans reported that reduced abundance *of Blautia* and members of the *Lachnospiraceae* family—both SCFA-producing taxa—was associated with an increased vulnerability to emotional/addictive eating. The same study found increased levels of *Anaeroplasma*, *Gastranaerophilales*, *Clostridiales vadinBB60*, and *Ruminococcaceae* in mice, exhibiting an addictive eating phenotype, which positively correlated with traits such as compulsivity, heightened motivation, and impaired control [29]. These findings suggest that gut microbiota may modulate eating behavior through pathways affecting motivation, reward processing, and emotional control.

### 3.3. Relationship Between Emotional Eating and Diet Quality in Subjects with Abdominal Obesity

#### 3.3.1. Definition of Diet Quality

Diet quality refers to the extent to which a diet provides all essential nutrients required to maintain good health, prevent disease, and promote overall well-being [123]. It is influenced by a combination of sociodemographic factors, lifestyle behaviors, dietary composition, health conditions, and cultural aspects [124].

A high-quality diet is characterized by the inclusion of a wide variety of nutrient-dense foods that align with dietary recommendations established by health authorities [123].

Assessing diet quality involves evaluating the extent to which an individual’s dietary intake conforms to current nutritional guidelines. This assessment relies on both quantitative and qualitative measures to estimate overall diet quality, examining the diversity of macronutrient and micronutrient intake as well as the presence of healthy or unhealthy dietary patterns [125]. These indicators consider aspects such as dietary variety, frequency of consumption, the presence of potentially harmful components, and lifestyle-related factors [125,126].

In clinical research, diet quality or dietary intake is commonly assessed using dietary indices. Instruments such as the Healthy Eating Index (HEI) [127], the Alternative Healthy Eating Index (AHEI), the Mediterranean Diet Score (MDS), the Diet Quality Index (DQI) [128], the DASH Diet Score [129], the Mean Adequacy Ratio (MAR) [130], and the Second Version of a Mini-Survey to Evaluate Food Intake Quality (Mini-ECCA v.2) [126] are among the validated tools that have been widely used across different studies and populations.

These indices provide valuable tools for researchers and health professionals to comprehensively assess diet quality in diverse contexts [128] and to identify overall dietary patterns.

#### 3.3.2. Effects of Emotional Eating on Diet Quality

Research evidence indicates a close relationship between EE, diet quality, and obesity, particularly AO. Low-quality diets—characterized by high consumption of sugar-sweetened beverages, fast food, baked goods, sweets, and salty snacks—have been associated with an increased risk of overweight, obesity, and AO, especially among men [131]. In contrast, greater dietary diversity, particularly higher intake of fruits, vegetables, nuts, dairy products, legumes, whole grains, and lean protein sources, has been linked to a lower likelihood of developing AO [132,133,134].

The impact of EE on unhealthy food consumption and AO has been supported by multiple studies [11,53,69]. Recent evidence indicates that EE scores are significantly higher in adults with obesity compared with individuals with a healthy BMI [135,136,137].

EE has been associated with excessive consumption of hyperpalatable foods rich in fats and sugars, which is linked to increases in BMI and abdominal circumference. Furthermore, longitudinal studies suggest that EE may mediate the relationship between depressive symptoms and increases in these anthropometric indicators over time [69].

Among individuals with AO, EE has been negatively associated with healthy dietary patterns and positively associated with the consumption of snacks and fast food [11,53]. Additionally, EE has been associated with higher intake of UPFs [36,37,38,40,42]. UPFs are widely available, relatively low-cost, and heavily promoted through mass media [135,138,139], which may facilitate their consumption across different populations. These products are generally characterized by high energy density, added sugars, unhealthy fats, and high palatability [20]. In this context, several studies have reported an association between negative emotional states, particularly stress and psychological distress, greater consumption of energy-dense foods and UPFs [140,141].

Together, these findings support an association between EE, emotional distress, and an unfavorable dietary pattern characterized not only by higher consumption of UPFs but also by lower intake of fruits, vegetables, and other fiber-rich foods [11,137]. This dietary profile is relevant because it may contribute to poorer diet quality, excess energy intake, and metabolic alterations that favor abdominal fat accumulation. However, the hypothesis that UPF intake mediates the relationship between EE and AO remains only partially supported by empirical evidence. Experimental evidence indicates that UPF-rich diets can increase ad libitum energy intake and body weight, while prospective studies have linked UPF intake with a higher risk of general and AO [142,143]. Therefore, UPFs may represent a plausible behavioral pathway through which EE contributes to excess energy intake and, potentially, AO. Nevertheless, longitudinal studies formally testing UPF intake as a mediator between EE and AO remain limited. Thus, the EE → UPFs → AO pathway should be interpreted as a plausible but still hypothetical mechanism that requires confirmation through prospective designs and mediation analyses.

Taken together, these findings underscore the importance of considering EE as a key behavioral factor in the development and maintenance of AO, as well as in the deterioration of diet quality.

### 3.4. Effects of Mindfulness and Mindful Eating on Emotional Eating and Abdominal Obesity

Mindfulness is defined as a temporary state of attention and awareness focused on the present moment, without judgment or reaction. Mindful eating emerges as a practical application of mindfulness. This practice may facilitate greater connection between the mind and body during the act of eating, and has been linked to increased awareness of hunger cues, satiety, and the emotions associated with food [144,145].

Standardized mindfulness-based interventions aim to cultivate a nonjudgmental awareness of present-moment experience [146]. Moreover, they have been shown to be effective in reducing subcortical responses to emotional stimuli [147], which may contribute to the reduction in EE behaviors. This effect is further supported by neuroimaging studies suggesting that mindfulness practices are associated with changes in neural circuits involved in reward processing, interoception, and appetite regulation, including the hypothalamus and insula, with potential implications in hunger and satiety signaling [148]. From a physiological perspective, mindfulness-based interventions may also downregulate the HPA axis, leading to reductions in cortisol levels and stress reactivity, both of which are key drivers of EE [149,150].

The evidence on mindfulness-based and mindful eating interventions shows more consistent effects on EE and related eating behaviors than on AO-related anthropometric outcomes (Table 3). Across the included studies, the term “statistically significant reduction” was used only when the original studies reported a significant *p* value, or an effect estimate with a confidence interval excluding the null value. Clinical relevance was interpreted separately, particularly for WC, because a statistically significant change does not necessarily indicate a clinically meaningful reduction in cardiometabolic risk.

Two cross-sectional observational studies provided associative evidence. In women with overweight or obesity, mindful eating moderated the relationship between emotional dysfunction, negative affect, and eating styles, including EE, with small but statistically significant moderation effects for emotional dysregulation × mindful eating on EE (R^2^ change = 0.03) and negative affect × mindful eating on EE (R^2^ change = 0.05) [151]. Similarly, in adults with obesity attending a clinical weight management service, mindful eating was negatively associated with EE (r = −0.592, *p* < 0.001) and external eating (r = −0.576, *p* < 0.001) [152]. Since these were cross-sectional studies, they do not provide evidence of intervention effects or changes in WC or body composition.

**Table 3:** Evidence of the effects of mindfulness and mindful eating on emotional eating and abdominal obesity.

Study Design	Population	Intervention/Exposure	Duration	Follow-Up	Comparator	Results on Emotional Eating	Effects on Waist Circumference/Anthropometry	Effect Size/Statistical Estimate	Reference
Cross-sectional observational study	151 women with overweight or obesity, Poland	No intervention was delivered. The main exposure/moderator was mindful eating,	NA	NA	NA	Mindful eating moderated the relationship between emotional dysfunction/negative affect and eating styles, including EE	Not assessed	Small significant moderation effects were observed for emotional dysregulation × mindful eating on EE (R^2^ change = 0.03) and negative affect × mindful eating on EE (R^2^ change = 0.05).	[151]
Cluster randomized controlled trial	76 adults with overweight or obesity in primary care, Spain	Mindful eating + treatment as usual	7 weeks	12-month after treatment.	Treatment as usual	Significant reduction in EE post-intervention and at 12 months	No significant changes in weight or physiological parameters	EE: post-treatment B = −0.27, *p* = 0.006, d = 0.35; 12-month follow-up B = −0.53, *p* < 0.001, d = 0.69. Effects were small post-treatment and moderate at follow-up.	[153]
Cross-sectional observational study	101 adults with obesity in a clinical weight management service, United Kingdom	No intervention was tested. The main exposures were self-compassion, mindfulness, and mindful eating	NA	NA	NA	Mindful eating was negatively associated with EE	Not assessed	Mindful eating showed a large negative correlation with EE (r = −0.592, *p* < 0.001) and external eating (r = −0.576, *p* < 0.001).	[152]
Prospective, longitudinal, experimental pre–post study.	82 individuals with obesity and binge eating disorder, Brazil	Eight individual mindful eating sessions plus non-calorie-restricted nutritional education workshops	8 weeks	Telephone follow-up 8 weeks after the intervention.	No control group	Significant reduction in binge eating episodes and BES scores	Significant reductions in weight, BMI and WC (*p* < 0.0001)	Effect sizes were NR. Statistical estimates were based on within-group Wilcoxon tests; all main outcomes showed *p* < 0.0001. At telephone follow-up, 71% reported maintaining weight loss and professional monitoring, while 29% reported returning to initial weight.	[154]
Randomized controlled trial with three parallel groups	138 women with class I and II obesity, Brazil	Three groups: ME + MER, mindful eating plus moderate energy restriction; MER, moderate energy restriction only; ME, mindful eating only.	6 months; 7 monthly sessions	No long-term post-intervention follow-up reported.	ME + MER vs. MER vs. ME.	EE decreased more in the ME group than in MER and ME + MER	Significant weight reduction in all groups, with no between-group differences	EE reduction favored ME alone: *p* < 0.001. Weight change: MER −3.9%, ME −3.3%, ME + MER −2.6%; *p* = 0.692. Waist circumference: MER −4.2%, ME −3.4%, ME + MER −3.6%; *p* = 0.844. Effect size NR.	[155]
Pragmatic randomized controlled trial with three arms	284 low-income women with overweight/obesity in primary health care, Brazil	Mindfulness program vs. mindful eating program vs. control	10 weeks	3 months	Control group and general mindfulness program	EE was not directly measured with a specific EE scale. However, binge eating severity and binge eating episodes decreased significantly in the mindful eating group	No significant changes in weight or most anthropometric outcomes	For binge eating, mindful eating program showed significant reductions vs. control post-intervention B = −4.27 to −5.03, *p* = 0.001–0.005; 3-month follow-up B = −5.38 to −5.85, *p* < 0.001–0.005. WC not significantly different; *p* = 0.482 reported for waist circumference comparison; effect size NR	[156]
Exploratory randomized controlled trial	61 inactive women with overweight/obesity, Denmark	Mindful eating vs. YogaDance vs. mindful eating + YogaDance vs. control	8 weeks	No post-intervention follow-up	Control group with no mindful eating or YogaDance intervention.	EE was not reported as a separate outcome. Eating behavior was assessed using the Intuitive Eating Scale-2 (IES-2). Mindful eating and mindful eating + YogaDance improved eating behavior and quality of life	Body weight decreased modestly in all intervention groups compared with control, but differences were not statistically significant. In complete-case analyses, WC showed modest reductions, especially in mindful eating and combined groups.	Eating behavior: Complete-case IES-2 improvement: mindful eating +0.5, *p* = 0.01, effect size = 0.91; combined +0.4, *p* = 0.01, effect size = 0.73. Body weight: Mindful eating −1.3 kg, *p* = 0.77, effect size = 0.15; YogaDance −3.0 kg, *p* = 0.48, effect size = 0.34; combined −1.8 kg, *p* = 0.69, effect size = 0.21. WC: mindful eating −3.9 cm, *p* = 0.06; combined −3.4 cm, *p* = 0.06.	[157]
Systematic review and meta-analysis	34 studies including adults with overweight or obesity	EE-focused interventions, including mindful eating, CBT and ACT	1 day–24 months	Follow-up varied or was not consistently reported	Treatment as usual, waitlist control, no control, or active psychological/behavioral comparison, depending on study.	EE decreased after intervention	Small reduction in body weight	Adjusted pooled effect: EE −2.37% −2.37% (95% CI: −3.76 to −0.99; I^2^ = 87.77%; *n* = 46 interventions); body weight: −1.08% (95% CI: −1.66 to −0.49; I^2^ = 64.65%; *n* = 37 interventions).	[158]
Systematic review and meta-analysis	14 intervention studies on mindful eating and cardiometabolic risk	Mindful eating interventions	4 to 24 weeks.	Follow-up varied across studies, from no follow-up to long-term follow-up up to 72 weeks in some trials.	Control groups included standard care, usual treatment, waiting list, psychoeducation, or interventions with the same structure but without mindful eating components.	EE was not pooled in the meta-analysis. However, individual studies reported significant improvements in EE/emotional hunger, including reductions at 10 weeks and at 6-month follow-up in some trials.	Body weight showed a significant reduction only at 12 months. WC showed no significant pooled effect at 3 months or 6 months.	Weight: 12-month pooled effect WMD −1.92 kg, 95% CI: −3.83 to −0.02. WC: 3 months WMD +0.49 cm, 95% CI: −1.11 to 2.09; 6 months WMD −0.78 cm, 95% CI: −2.18 to 0.63.	[159]

ACT, Acceptance and Commitment Therapy; BES, Binge Eating Scale; BMI, body mass index; CI, confidence interval; CBT, cognitive behavioral therapy; EE, emotional eating; ME, mindful eating; MER, moderate energy restriction; NA, not applicable; NR, not reported; NS, non-significant; WC, waist circumference; WMD, weighted mean difference. Effect sizes are presented as reported in the original studies; when not available, the direction of the effect or statistical significance was described.

Intervention studies reported more consistent changes in EE than in anthropometric outcomes. A 7-week cluster randomized controlled trial in adults with overweight or obesity showed statistically significant reductions in EE after mindful eating plus usual care, both post-treatment (B = −0.27, *p* = 0.006, d = 0.35) and at 12-month follow-up (B = −0.53, *p* < 0.001, d = 0.69), indicating small effects immediately after treatment and moderate effects at follow-up [153]. However, no statistically significant changes were reported regarding weight or physiological parameters. In another randomized controlled trial with three parallel groups, levels of EE decreased more in the mindful eating group than in the moderate energy restriction and combined groups (*p* < 0.001), whereas weight and WC decreased in all groups without significant between-group differences [155]. Therefore, the anthropometric changes in this trial should be interpreted as non-superiority between interventions rather than as a specific effect of mindful eating.

A pragmatic randomized controlled trial conducted in primary care reported improvements in binge eating severity and binge eating episodes in the mindful eating group compared with control, with significant effects post-intervention and at 3-month follow-up. However, EE was not reported separately as an independent EE subscale, and no significant changes were found in weight or most anthropometric outcomes [156]. Likewise, an exploratory randomized controlled trial evaluated mindful eating, YogaDance, their combination, and control group, found improvements in eating behavior measured with the Intuitive Eating Scale-2 in complete-case analyses, but EE was not reported as a separate outcome [157]. In this study, body weight decreased numerically in all intervention groups compared with control, but differences were not statistically significant. WC also showed modest reductions in complete-case analyses, particularly in mindful eating and combined groups, but these changes did not reach statistical significance (mindful eating: −3.9 cm, *p* = 0.06; combined intervention: −3.4 cm, *p* = 0.06) [157].

One prospective pre–post study in individuals with obesity and binge eating disorder reported statistically significant within-group reductions in binge eating episodes, Binge Eating Scale scores, weight, BMI, and WC after an 8-week mindful eating intervention [154]. WC decreased from 103.5 to 100.5 cm (*p* < 0.0001), corresponding to an absolute reduction of 3.0 cm [154]. This magnitude may be clinically relevant if sustained, because WC is an indicator of abdominal adiposity and cardiometabolic risk. However, because this study lacked a control group and effect sizes were not reported, the clinical significance and causal attribution of this WC change should be interpreted cautiously.

The systematic reviews and meta-analyses support these findings. A meta-analysis of 34 studies on interventions targeting EE reported a statistically significant adjusted reduction in EE scores of −2.37% (95% CI: −3.76 to −0.99; I^2^ = 87.77%; 46 interventions) and a small adjusted reduction in body weight of −1.08% (95% CI: −1.66 to −0.49; I^2^ = 64.65%; 37 interventions) [158]. Another systematic review and meta-analysis focused specifically on mindful eating and cardiometabolic risk factors found that EE was not pooled as a meta-analytic outcome, although individual studies reported reductions in EE or emotional hunger [156]. In this review, body weight showed a statistically significant pooled reduction only at 12 months (WMD = −1.92 kg; 95% CI: −3.83 to −0.02), while WC did not show significant pooled reductions at 3 months (WMD = +0.49 cm; 95% CI: −1.11 to 2.09) or 6 months (WMD = −0.78 cm; 95% CI: −2.18 to 0.63) [159].

Overall, the results indicate that mindfulness-based and mindful eating interventions are associated with statistically significant improvements in EE, binge eating, and eating behavior outcomes, whereas their effects on AO-related anthropometric indicators are less consistent. Reductions in WC were statistically significant in some individual studies, but pooled effects were not significant. Therefore, although WC reductions of approximately 3 cm may be potentially clinically meaningful when sustained, current evidence does not yet demonstrate a consistent clinically relevant effect of mindful eating interventions on OA.

## 4. Discussion

Recent studies have highlighted a bidirectional relationship between mood disorders and obesity [160,161]. EE and AO appear to be interconnected through complex psychological, physiological, behavioral, and sociocultural mechanisms [11,19,30,53]. Obesity has been associated with impaired emotion regulation, chronic stress, anxiety, and depressive symptoms, all of which may contribute to EE behaviors [19,53,57]. Conversely, emotional distress may trigger EE even in individuals without obesity [11,15,16,30], potentially leading to increased energy intake, weight gain, and subsequent accumulation of abdominal fat [39,41,69]. These behaviors often involve the consumption of highly palatable, energy-dense foods, thereby perpetuating a cycle of emotional distress and body fat gain.

From a psychological and neuroendocrine perspective, chronic stress, anxiety, and depressive symptoms may activate the HPA axis, increasing cortisol and glucocorticoid levels, which can promote abdominal fat deposition and stimulate the intake of comfort foods rich in fat and sugar [19,43,44,58,60]. Sustained activation of stress-related pathways may therefore reinforce emotionally driven eating behaviors and contribute to the development and maintenance of AO [19,58,60,68]. Nevertheless, this pathway remains complex, as some studies have not identified significant associations between cortisol levels and dysregulated eating behaviors [162,163]. These inconsistent findings do not necessarily contradict the involvement of the HPA axis; rather, they suggest that the relationship between cortisol, EE, and AO may be context-dependent.

Several methodological and biological factors may explain this variability. Cortisol follows a marked diurnal rhythm and shows a cortisol awakening response [164], making its interpretation highly dependent on awakening time, sampling time, and the number of samples collected [164,165]. Therefore, studies relying on single-point cortisol measurements may fail to capture the dynamic activity of the HPA axis, particularly when sampling protocols do not account for circadian variation or daily fluctuations in cortisol secretion [162]. In addition, different assessment methods, such as salivary, serum, urinary, or hair cortisol, reflect different temporal windows of cortisol exposure [165], which may limit comparability across studies. Individual variability in stress responsivity and stressor chronicity may also modify HPA-axis activity, since chronic stress is not always associated with uniformly elevated cortisol levels and may instead produce variable or blunted cortisol patterns over time [163]. This variability may be particularly relevant in obesity, where adiposity itself has been associated with alterations in cortisol activity and HPA-axis regulation [166]. Thus, heterogeneous findings should be interpreted cautiously, as they may reflect methodological and biological variability rather than the absence of a neuroendocrine contribution to EE and AO.

Physiological dysregulation may also represent a key link between EE and AO. AO is characterized by a chronic low-grade inflammatory state that disrupts hormonal signaling and contributes to insulin resistance and metabolic dysfunction [7]. Some studies have reported associations between inflammatory biomarkers, such as IL-6 and C-reactive protein (CRP), and obesity-related eating behaviors, including loss of control over food intake [21]. These inflammatory alterations may, in turn, contribute to EE behaviors. However, the number of studies addressing this relationship remains limited.

In addition, alterations in neurotransmitter systems and appetite-regulating hormones—including dopaminergic and serotonergic pathways, ghrelin, and leptin—have been identified as potential contributors to reward-driven eating, appetite dysregulation, and adiposity-related metabolic alterations [58,60,78,91,93,96,161]. These neuroendocrine pathways may also intersect with mechanisms involved in visceral adiposity and metabolic risk [7,85]. However, many mechanistic findings derive from experimental or preclinical models, and their extrapolation to human populations should therefore be approached with caution [74,75,87,88,89,90,93]. Another emerging area of interest is the gut–brain axis. Imbalances in gut microbiota composition, including changes in the *Firmicutes*-to-*Bacteroidetes* ratio and alterations in short-chain fatty acid–producing bacteria, have been suggested to modulate appetite and eating behavior through neuroendocrine, neurotransmitter-related, and inflammatory pathways [27,104,106,107,108,109,112,113,114,115,118]. Although this field has shown promising results, evidence remains preliminary, and well-controlled longitudinal studies are needed to elucidate causality and identify specific mechanisms.

From a behavioral perspective, EE has been associated with low-quality dietary patterns characterized by higher consumption of UPFs, fast food, added sugars, and saturated fats, along with lower intake of fruits, vegetables, and dietary fiber [11,36,40,53,137]. This relationship may represent a pathway through which EE indirectly contributes to AO. However, the directionality of this association remains unclear, as EE and poor diet quality may mutually reinforce each other within a dysfunctional cycle. Similarly, hedonic food-seeking behavior has been associated with higher BMI and EE, although reported effect sizes are generally small [25]. Hedonic hunger has also been linked to emotional eating and difficulties in emotion regulation, suggesting that emotional dysregulation may partly explain the relationship between hedonic food motivation, EE, and weight-related outcomes [22,38,53,55]. However, longitudinal studies are still needed to confirm these associations and clarify their temporal direction.

The available evidence suggests that EE may be associated with AO and related anthropometric indicators, such as WC or waist-to-height ratio, although the strength of this association varies across populations and study designs [11,51,135,137]. However, a clear causal relationship cannot yet be established, given the complexity of the mechanisms involved and the influence of multiple interpersonal, biological, environmental, and sociocultural factors. Cultural norms related to food practices, emotional expression, body image, gender roles, and access to hyperpalatable foods may shape eating behaviors differently across populations [124,138,139,141]. For example, associations between eating behaviors and anthropometric indicators appear to vary according to age, sex, and population characteristics in European samples, while studies in other adult populations have also reported positive associations between emotional or external eating styles and obesity-related indicators [12,40,49,51,52]. Cross-cultural differences in dietary patterns, stress exposure, urbanization, and stigma surrounding obesity and mental health may therefore contribute to heterogeneity in the observed findings.

Interpretation of the available evidence should also consider substantial methodological heterogeneity across studies. Included investigations differed considerably in study design, sample characteristics, psychometric instruments used to assess EE, anthropometric indicators of AO, and adjustment for potential confounding variables. Moreover, most studies were cross-sectional, which limits causal inference and temporal interpretation of the relationship between EE and AO. Variability in EE assessment tools may additionally explain inconsistencies in reported associations across populations. Consequently, caution is warranted when generalizing results, and future studies should prioritize culturally sensitive approaches, standardized methodologies, and longitudinal designs.

Regarding intervention strategies, practices such as mindful eating have demonstrated positive effects in reducing EE and improving eating behavior [151,154,156,158,159], even in the absence of strict caloric restriction [151]. Although some studies have reported modest improvements in WC [159], anthropometric outcomes remain inconsistent, and these interventions require further validation across diverse populations and contexts.

In summary, although current evidence supports a potential bidirectional association between EE and AO, substantial gaps remain in the understanding of the underlying mechanisms. EE and AO should be interpreted as part of a multidimensional and context-dependent relationship in which biological, psychological, behavioral, and sociocultural factors interact dynamically. High-quality longitudinal and experimental studies incorporating neuroendocrine, immunological, microbiota-related, behavioral, and psychosocial variables are needed to elucidate causal pathways and inform the development of more precise and effective intervention strategies.

Taken together, the available evidence supports a multidimensional and context-dependent relationship between EE and AO, in which biological, psychological, behavioral, and sociocultural factors interact dynamically.

## 5. Conclusions

The available evidence suggests that EE may represent a potential risk factor in the development and maintenance of AO. In human studies, this relationship has been mainly supported by observational evidence linking EE with psychological mechanisms, such as stress, anxiety, depressive symptoms, and difficulties in emotional regulation, as well as with poorer diet quality, higher intake of energy-dense foods and UPFs, increased BMI, WC, and metabolic dysregulation. Human evidence also suggests the potential involvement of physiological pathways, including HPA-axis activity, low-grade inflammation, appetite-regulating hormones, and gut–brain axis alterations; however, these findings remain heterogeneous and are mostly associative.

In contrast, preclinical studies in rodent models have provided more detailed mechanistic evidence regarding the neuroendocrine and metabolic pathways that may link stress-related eating behaviors with adiposity. These studies suggest that chronic stress, HPA-axis activation, glucocorticoid exposure, reward-related signaling, appetite-regulating neuropeptides, hypothalamic inflammation, and gut microbiota alterations may contribute to hyperphagia, preference for highly palatable foods, sustained positive energy balance, and visceral fat accumulation. Therefore, evidence from animal models supports the biological plausibility of the EE–AO relationship, but these findings should be interpreted as complementary mechanistic evidence rather than direct confirmation of causality in humans.

Additionally, hedonic food seeking has emerged as a relevant component in this interaction. In human studies, hedonic food seeking has been associated with both higher levels of EE and increased BMI, particularly among individuals with difficulties in emotional regulation. These findings suggest that EE may contribute to impulsive eating behaviors and a sustained positive energy balance, thereby favoring visceral fat accumulation. However, the magnitude of the reported effects is modest, and the current evidence does not yet allow the establishment of robust causal relationships, highlighting the need for more rigorous longitudinal and experimental research.

EE is also associated with poorer diet quality, characterized by higher consumption of UPFs, added sugars, fats, fast food, and energy-dense foods. This association may be partly explained by impulsive intake and neuroendocrine systems involved in appetite regulation, stress response, reward processing, and emotional regulation. Nevertheless, because some of these mechanisms are primarily derived from preclinical models, whereas human evidence remains largely observational, the EE → neuroendocrine dysregulation → poorer diet quality → AO pathway should be interpreted as biologically plausible but not fully established.

Furthermore, although growing evidence supports the involvement of cortisol, inflammatory markers, and the gut–brain axis in the relationship between EE and AO, these findings remain heterogeneous and are not yet fully conclusive. Overall, although the literature supports a probable association between EE and AO, a clear causal relationship cannot yet be established due to methodological heterogeneity, modest effect sizes, and the predominance of cross-sectional designs.

Another important consideration is that improvements in eating-related psychological outcomes may not immediately translate into physiological or anthropometric changes. Mindfulness-based and mindful eating interventions may help reduce EE, food cravings, stress-related eating, and loss-of-control eating by improving emotional regulation and awareness of hunger and satiety cues. However, these changes do not necessarily imply a sustained negative energy balance, which is required to reduce body weight, WC, or metabolic risk markers. This may be particularly relevant when interventions are short in duration, do not include an explicit caloric restriction component or structured dietary prescription, or when adherence to the protocol is low. Therefore, improvements in EE and related psychological outcomes may represent an early behavioral response, whereas changes in AO may require longer intervention periods, higher adherence, and sustained modifications in energy intake, diet quality, physical activity, sleep, and metabolic regulation.

Overall, although the literature supports a probable association between EE and AO, a clear causal relationship cannot yet be established due to methodological heterogeneity, modest effect sizes, and the predominance of cross-sectional designs. Therefore, future longitudinal studies should follow individuals with pronounced EE over time to determine whether EE precedes the development of AO, reflected by progressive increases in WC, visceral adiposity, and metabolic risk, or whether AO itself contributes to greater emotional distress and subsequent maladaptive eating behaviors. Clarifying this temporal sequence is essential to distinguish cause from consequence and to identify potential bidirectional pathways between emotional regulation, diet quality, neuroendocrine dysregulation, and abdominal fat accumulation.

## Figures and Tables

**Table 1:** Association between emotional eating and abdominal obesity.

Methodology	Study Population	Main Findings	Reference
Prospective cohort study with a 7-year follow-up. Depressive symptoms, EE, and changes in WC were assessed.	5024 Finnish adults	EE appeared to mediate the association between depressive symptoms and 7-year changes in WC (R^2^ = 0.045), suggesting a possible longitudinal link between depressive symptoms and abdominal adiposity.	[13]
Cross-sectional study assessing EE and anthropometric indices, including WC.	3742 Turkish adults	Positive associations were observed between EE and WC, body weight, BMI, and WHtR (*p* < 0.05). Individuals with higher metabolic risk based on WC also showed higher EE scores.	[50]
Cross-sectional study assessing EE, binge eating, and anthropometric measures.	78 Slovenian adults	EE was positively associated with WC and AO. In regression analyses, EE and BMI were associated with binge eating behavior and AO.	[12]
Cross-sectional study assessing EE and anthropometric parameters, including WC.	328 Greek adults	Overall, EE showed a negative association with WC and BMI; however, this association varied by age and appeared to become positive among participants older than 35.9 years.	[49]
Cross-sectional study assessing EE, BMI, and WHtR.	353 Polish university students	In women, higher EE was associated with higher BMI (r = 0.184; *p* = 0.008). In men, higher EE was associated with higher WHtR (*p* = 0.037).	[51]
Cross-sectional study assessing EE, external eating, and dietary restraint. BMI and WC were measured.	200 Algerian adults	Individuals with obesity reported higher levels of EE and external eating than those with normal BMI. Regression analyses suggested positive associations between EE, external eating, BMI, and WC (*p* < 0.05).	[52]

EE, emotional eating; WC, waist circumference; BMI, body mass index; WHtR, waist-to-height ratio; AO, abdominal obesity; R^2^, coefficient of determination.

**Table 2:** Neurotransmitters and hormones: their relationship with emotional eating and abdominal obesity.

Neurotransmitter/Hormone	Main Mechanisms	Connection with EE	Connection with AO	Evidence Level	References
POMC/α-MSH	Activates MC4R → catabolic pathways → ↓ food intake and ↑ thermogenesis. Mitochondrial dysfunction impairs appetite and energy expenditure regulation.	Chronic stress hyperactivates POMC neurons → anhedonia, hopelessness → EE.	↓ CSF POMC levels correlated with ↑ body fat; dysfunction and insulin resistance.	Animal + human	[71,72,73,74]
NPY	Stimulates appetite via NPY1R/NPY5R; ↓ energy expenditure and ↑ adipogenesis.	Stress increases NPY → emotional intake of hyperpalatable foods.	↑ NPY in obesity → ↑ appetite, ↓ thermogenesis, and ↑ fat accumulation.	Mainly animal	[75,76,77,78]
AgRP	Reduces energy expenditure, inhibits aversive signaling in the parabrachial nucleus, ↑ motivation for energy-dense foods.	Reinforces intake of rewarding foods as an emotional response.	Promotes fat accumulation and emotion-related food learning.	Mainly animal	[79,80,81,82,83]
Glutamate	↑ Circulating glutamate correlated with ↑ abdominal fat and ↓ adiponectin.	Preclinical evidence suggests that glutamatergic circuits may contribute to stress-related compulsive eating-like behaviors.	↑ Plasma glutamate in obesity and type 2 diabetes.	Animal + human	[84,85,86,87]
GABA	Inhibits weight gain, suppresses adipogenesis, and promotes lipolysis; modulates energy balance.	GABA activation in the VTA → anxiety-like behavior and overconsumption of hyperpalatable foods.	Improves lipid profile and promotes browning of adipose tissue in obesity.	Animal studies	[88,89,90]
Serotonin (5-HT)	Activates 5-HT2C receptors in POMC neurons → regulates appetite and energy expenditure.	↓ Serotonergic signaling → ↓ appetite control and ↑ emotionally driven food seeking.	↓ 5-HT in obesity → ↑ intake of rewarding foods and abdominal fat accumulation.	Animal + human	[23,24,91]
Oxytocin	Activates POMC and inhibits AgRP/NPY neurons → promotes satiety; ↓ motivation for palatable foods; enhances thermogenesis and lipolysis.	Modulates emotional control of intake, reducing emotionally driven food seeking.	Reduces visceral fat and promotes browning of white adipose tissue.	Animal + human	[92,93,94,95]
Leptin	Inhibits orexigenic neurons and activates anorexigenic neurons; ↓ motivation for rewarding foods.	Leptin resistance, exacerbated by stress, facilitates EE.	Leptin resistance in obesity → overeating and ↑ visceral fat.	Animal + human	[58,96,97]
Ghrelin	Activates AgRP/NPY neurons; ↑ dopamine release in reward circuits; activates AMPK, SIRT1/p53, CaMK1D, and mTOR pathways.	Under stress, counteracts HPA axis-induced appetite suppression → promotes EE.	↑ Food intake, ↓ energy expenditure → body fat accumulation.	Animal + human	[58,98,99,100]
Insulin	Inhibits AgRP/NPY and activates POMC neurons; ↓ motivation for palatable foods via reward pathways.	Central insulin resistance (e.g., in the central amygdala) impairs appetite control → facilitates EE.	Central insulin resistance → ↑ visceral fat accumulation.	Mainly animal	[81,101,102,103]

5-HT, 5-hydroxytryptamine; AgRP, agouti-related peptide; AO, abdominal obesity; AMPK, AMP-activated protein kinase; CaMK1D, calcium/calmodulin-dependent protein kinase 1D; CSF, cerebrospinal fluid; EE, emotional eating; GABA, gamma-aminobutyric acid; HPA, hypothalamic–pituitary–adrenal; MC4R, melanocortin-4 receptor; mTOR, mechanistic target of rapamycin; NPY, neuropeptide Y; NPY1R/NPY2R/NPY5R, neuropeptide Y receptors; POMC, pro-opiomelanocortin; SIRT1, sirtuin 1; VTA, ventral tegmental area. Symbols: → indicates a proposed direction of association or pathway; ↑ indicates increased expression, activation, concentration, or activity; ↓ indicates decreased expression, activation, concentration, or activity.

## Data Availability

No new data were created or analyzed in this study. Data Sharing is not applicable to this article.

## Abbreviations

The following abbreviations are used in this manuscript:

5-HT	5-hydroxytryptamine
ACT	Acceptance and Commitment Therapy
AgRP	Agouti-related peptide
AHEI	Alternative Healthy Eating Index
AMPK	AMP-activated protein kinase
AO	Abdominal obesity
AT	Adipose tissue
BED	Binge eating disorder
BES	Binge Eating Scale
BMI	Body Mass Index
BSQ	Body Shape Questionnaire
CBT	Cognitive behavioral therapy
CNS	Central nervous system
Crif1	CR6-interacting factor 1
CRP	C-reactive protein
DASH	Dietary Approaches to Stop Hypertension
DQI	Diet Quality Index
EE	Emotional eating
FFAR2	Free fatty acid receptor 2
FFAR3	Free fatty acid receptor 3
FFA	Free fatty acids
FXR	Farnesoid X receptor
GABA	Gamma-aminobutyric acid
GAD	Generalized anxiety disorder
GLP-1	Glucagon-like peptide-1
GPR	G protein-coupled receptor
HEI	Healthy Eating Index
HPA	Hypothalamic–Pituitary–Adrenal axis
IL-6	Interleukin-6
ITT	Intention-to-treat
LOC eating	Loss of control eating
LPS	Lipopolysaccharide
MAR	Mean Adequacy Ratio
MB-EAT	Mindfulness-Based Eating Awareness Training
MB-EAT-SP	Mindfulness-Based Eating Awareness Training—Spanish adapted version
MBHP	Mindfulness-Based Health Promotion
MC4R	Melanocortin 4 receptor
MDS	Mediterranean Diet Score
ME	Mindful eating
MFN1	Mitofusin 1
MFN2	Mitofusin 2
Mini-ECCA v.2	Mini-Survey to Evaluate Food Intake Quality, version 2
mTOR	Mechanistic target of rapamycin
NAc	Nucleus accumbens
NHLBI	National Heart, Lung, and Blood Institute
NPY	Neuropeptide Y
NPY1R	Neuropeptide Y receptor 1
NPY2R	Neuropeptide Y receptor 2
NPY5R	Neuropeptide Y receptor 5
OPA1	Optic atrophy 1
POMC	Pro-opiomelanocortin
PP	Per protocol
PYY	Peptide YY
RCT	Randomized controlled trial
ROS	Reactive oxygen species
SCFAs	Short-chain fatty acids
TLRs	Toll-like receptors
TNF-α	Tumor necrosis factor alpha
UCP2	Uncoupling protein 2
VTA	Ventral tegmental area
WC	Waist circumference
WHOQOL-BREF	World Health Organization Quality of Life—Brief version
